# Aesthetic interventions in immune-mediated diseases: integrating safety perceptions and quality-of-life-driven care

**DOI:** 10.3389/fimmu.2026.1786267

**Published:** 2026-04-16

**Authors:** Shiuan-Tzuen Su, James C.-C. Wei

**Affiliations:** 1 Department of Surgery, Chung Shan Medical University Hospital, Taichung, Taiwan; 2 School of Medicine, Chung Shan Medical University, Taichung, Taiwan; 3 Institute of Medicine, Chung Shan Medical University, Taichung, Taiwan; 4 Division of Allergy, Immunology and Rheumatology, Department of Internal Medicine, Chung Shan Medical University Hospital, Taichung, Taiwan; 5 Graduate Institute of Integrated Medicine, China Medical University, Taichung, Taiwan

**Keywords:** cosmetic interventions, patient-reported outcomes, quality-of-life, rheumatology, safety

## Introduction

Immune-mediated diseases (IMDs), including systemic lupus erythematosus (SLE), rheumatoid arthritis (RA), systemic sclerosis, Sjögren’s syndrome, and other autoimmune connective tissue diseases (CTDs), are chronic conditions characterized by persistent inflammation, multisystem involvement, and fluctuating disease activity. These disorders often manifest during early to middle adulthood and are commonly accompanied by chronic pain, fatigue, cutaneous changes, scarring, and visible physical alterations that profoundly affect body image, self-esteem, and psychosocial well-being (1). This opinion argues that aesthetic procedures in patients with IMDs require individualized, immunologically informed risk assessment and interdisciplinary shared decision-making (SDM) to ensure safety and optimize clinical outcomes.

A growing body of evidence has demonstrated that IMDs exert profound and persistent effects on quality of life (QoL), encompassing physical function, psychological well-being, social participation, and work productivity (2, 3). Symptoms such as chronic pain, fatigue, sleep disturbances, and mood changes frequently persist even when objective inflammatory markers suggest adequate disease control. Clinician-assessed disease activity often differs from patients’ perceived health, underscoring the importance of patient-centered assessment. Evidence from large cross-sectional web-based surveys indicates that low self-esteem was highly prevalent among patients with IMDs, particularly among women and was strongly associated with poorer patient global assessment and depressive symptoms. Notably, a substantial proportion of patients report interest in aesthetic or cosmetic procedures, motivated primarily by the desire to improve self-image and QoL rather than to modify disease activity itself (1).

## Growth of aesthetic and minimally invasive cosmetic procedures

### Existing evidence on safety

Over the past decade, aesthetic and minimally invasive cosmetic procedures, such as dermal fillers, laser-based treatments, and minor plastic surgical interventions, have expanded rapidly worldwide. Patients with IMDs have increasingly sought these procedures to address disease-related cutaneous manifestations, scarring, and appearance-related concerns. As summarized in **Table 1**, current evidence on aesthetic procedures in patients with IMDs is primarily based on observational designs, including cross-sectional surveys (1), single-center retrospective study (4), and administrative database analyses (5). These approaches are vulnerable to selection bias, residual confounding, and underreporting of late complications. The absence of randomized controlled trials limits causal inference and weakens the strength of safety recommendations.

**Table 1:** Clinical application of aesthetic procedures in immune-mediated diseases.

Author (Year)	Participants	Disease context	Procedure type	Key findings	Study design/level of evidence
Maciel ALF et al., 2026 (1)	1665	IMRD	Aesthetic plastic surgery (survey-based)	Optimized disease control and mental health support were crucial for safe decision-making	Cross-sectional/III
Felis-Giemza A et al.2024 (4)	497	AIRD	Mixed aesthetic procedures	Procedures were well tolerated with high satisfaction when performed during disease remission	Cross-sectional/III
Rubio GA et al.2018 (5)	537	CTDs	Abdominoplasty	Feasible, but associated with increased risks of VTE and blood transfusion	Retrospective/II
Rubio GA et al.2019 (18)	830	CTDs	Autologous breast reconstruction (flap)	Feasible in CTDs patients, but carried higher risks of wound and medical complications	Retrospective/II
Abbas F et al.2024 (20)	638	CTDs	Various aesthetic surgery	Generally safe in CTDs patients; breast augmentation required careful preoperative counseling	Retrospective/II
Koren A et al.2022 (23)	194	AIIRD	Dermal filler injections	Non-permanent fillers were safe in well-controlled AIIRDs; avoided permanent fillers or active disease	Cross-sectional/III

IMRD, immune-mediated rheumatic diseases; AIRD, autoimmune inflammatory rheumatic diseases; CTDs, connective tissue diseases; VTE, venous thromboembolism; AIIRD, autoimmune inflammatory rheumatic diseases.

The included studies were predominantly observational in design. Conclusions regarding procedural safety should be interpreted cautiously in the absence of randomized controlled trials.

Observational studies suggest that in carefully selected patients with stable or low disease activity, aesthetic procedures can be safely performed with low complication rates (4, 6). As disease control improves, patients increasingly seek cosmetic interventions to address aging-related or disease-associated changes, including skin atrophy, facial asymmetry, scarring, and corticosteroid-induced lipodystrophy. Laser and botulinum toxin treatments can be safely performed in patients with IMDs when disease activity is well controlled (7, 8). Complication rates were comparable to the general population, with mostly mild and self-limited effects.

The literature highlights substantial heterogeneity in risk associated with aesthetic procedures, largely determined by the type of material and technique used. Injections involving exogenous or permanent materials, particularly hyaluronic acid–based fillers and biostimulatory agents, have been linked to delayed inflammatory reactions, granulomatous responses, and immune-mediated adverse events in susceptible individuals. Case reports and observational studies suggest that underlying autoimmune disease, immune dysregulation, and biologic therapy may modify both risk and clinical presentation of these complications (9). Such reactions may occur months or even years after the initial procedure and include rare entities such as autoimmune/autoinflammatory syndrome induced by adjuvants (ASIA) (10). ASIA remains a debated construct. Although exposure to implants, fillers, or vaccines have been temporally associated with immune-mediated symptoms (11), diagnostic criteria rely largely on clinical patterns and exclusion of alternative causes (10). Overlap with established autoimmune diseases and heterogeneous presentations complicate attribution (12). Thus, ASIA should be approached cautiously, emphasizing rigorous differential diagnosis and individualized clinical judgment.

Early postoperative monitoring should include close assessment of local inflammation (pain, swelling, induration) and systemic symptoms such as fever, arthralgia, or fatigue. Clinicians should use serial clinical assessments, basic inflammatory markers, autoantibody screening, and magnetic resonance imaging (MRI) when symptoms persist or progress. ASIA should be considered with prior filler exposure, especially with delayed onset or progressive inflammation. These findings highlight the importance of careful patient selection, strict aseptic technique, and adapted injection strategies aimed at minimizing tissue trauma and inflammation, which may otherwise precipitate disease flares or chronic inflammatory responses.

### Immunological considerations

Nevertheless, the immunologic context remains critical. Laser and energy-based devices cause controlled tissue injury and trigger wound healing processes that depend on well-regulated immune signaling.

In SLE, immune dysregulation is characterized by sustained type I interferon (IFN-α) activation driven by plasmacytoid dendritic cells, immune complex–mediated Toll-like receptor (TLR7/9) signaling, and impaired clearance of apoptotic debris and neutrophil extracellular traps (NETs), amplifying autoantibody production and systemic inflammation (13, 14). In contrast, RA is primarily driven by synovial macrophages, fibroblast-like synoviocytes, and T cells, with cytokines such as interleukin-6 (IL-6), tumor necrosis factor-α (TNF-α), and IL-17 promoting chronic synovitis, pannus formation, and receptor activator of nuclear factor-kB ligand (RANKL)-mediated osteoclast activation leading to bone erosion (15, 16). While SLE reflects systemic loss of tolerance with immune complex deposition, RA represents localized cytokine-driven joint inflammation. These mechanistic differences may influence immune responses to aesthetic procedures and the risk of inflammatory complications.

In patients with autoimmune diseases or those receiving immunosuppressive therapy, altered inflammatory and repair responses might influence both treatment efficacy and safety. Therefore, pre-procedure assessment should consider disease activity, current immunomodulatory treatment, and any history of impaired wound healing or hypersensitivity reactions. Foreign dermal fillers may act as immune stimuli that activated innate immunity, leading to neutrophil recruitment, macrophage activation, and, in some cases, delayed foreign-body granuloma formation. These reactions might be enhanced by infection, biofilm formation, or immune memory at the injection site.

While immunosuppressive therapies broadly reduce lymphocyte activity and proinflammatory cytokine production, potentially lowering the risk of delayed hypersensitivity, immunomodulatory and biologic agents selectively altered immune pathways and might increase susceptibility to delayed inflammatory complications (7). Together, these mechanisms highlight the importance of individualized immune assessment when planning aesthetic procedures in patients with IMDs.

The expanding use of aesthetic procedures among patients with IMDs reflects improved disease control and growing attention to QoL concerns. These findings highlight the importance of individualized risk assessment, careful patient selection, and immunologically informed procedural planning to optimize outcomes and minimize immune-mediated complications.

## Importance of patient-reported outcomes and safety perceptions

Aesthetic procedures, ranging from minimally invasive injectables to energy-based dermatologic interventions, are increasingly sought by patients with IMDs. Traditional assessments of aesthetic procedure safety have primarily focused on clinician-reported complications, such as infection, tissue necrosis, wound dehiscence, or thromboembolic events. While these outcomes are clinically meaningful, they do not fully capture the patient experience. Increasing attention has therefore been directed toward PROs (17), including satisfaction with appearance, psychosocial confidence, emotional distress, and perceived QoL. Patients with IMDs often report or experience heightened concerns regarding infection risk, impaired wound healing, autoimmune flare activation, and potential interactions between aesthetic procedures and ongoing immunosuppressive therapies.

Single-center surveys of patients with IMDs suggest that most patients reported satisfaction with aesthetic procedures and expressed willingness to undergo repeat treatments, even when mild adverse events occurred (4). Importantly, patient-reported adverse events were often transient and localized; however, they exerted a disproportionate influence on patients’ perceptions of safety, anxiety regarding disease exacerbation, and trust in healthcare providers. Furthermore, studies on ASIA syndrome emphasize that inadequate pre-procedure counseling and limited understanding of immune-related risks significantly contributes to negative safety perceptions and long-term dissatisfaction (10) Autologous breast reconstruction, including free flap procedures, appears feasible in patients with autoimmune CTDs. Although microvascular complication rates are comparable to those without CTDs, these patients demonstrate higher risks of wound complications, pulmonary events, and venous thromboembolism (18). Collectively, these findings indicate that PROs and safety perceptions represent distinct and essential dimensions of outcome assessment, complementing objective clinical endpoints. To operationalize these dimensions, a proposed PROs checklist tailored to aesthetic procedures in IMDs is presented (**Table 2**).

**Table 2:** Proposed patient-reported outcomes checklist for aesthetic procedures in immune-mediated diseases.

Domain	Item	Question	Scale
Appearance Satisfaction	Q1	Overall satisfaction with appearance	0–10
Appearance Satisfaction	Q2	Natural look of treated area	0–10
Appearance Satisfaction	Q3	Match with expectations	0–10
Appearance Satisfaction	Q4	Comfort showing treated area socially	0–10
Psychosocial Benefit	Q5	Improved self-confidence	0–10
Psychosocial Benefit	Q6	Increased social engagement	0–10
Psychosocial Benefit	Q7	Emotional improvement	0–10
Psychosocial Benefit	Q8	Reduced appearance-related distress	0–10
Safety Perception	Q9	Concern about infection	0–10
Safety Perception	Q10	Concern about wound healing	0–10
Safety Perception	Q11	Concern about disease flare	0–10
Safety Perception	Q12	Concern about interaction with immunotherapy	0–10
Symptom Burden	Q13	Pain or burning sensation	0–10
Symptom Burden	Q14	Swelling or foreign body sensation	0–10
Symptom Burden	Q15	Pigmentation or redness impact	0–10
Symptom Burden	Q16	Impact on daily activities	0–10
Global Satisfaction	Q17	Overall satisfaction	5-point Likert
Global Satisfaction	Q18	Willingness to repeat procedure	5-point Likert
Global Satisfaction	Q19	Willingness to recommend	5-point Likert

The proposed Patient-Reported Outcomes Checklist for Aesthetic Procedures in immune-mediated diseases was conceptually developed based on established aesthetic and reconstructive instruments, including the BREAST-Q and FACE-Q, which assessed appearance satisfaction, psychosocial well-being, and treatment outcomes. Domains were adapted to reflect immune-specific safety concerns, such as fear of infection, impaired wound healing, and disease flare. The use of 0–10 numeric rating scales allows greater sensitivity in capturing safety perceptions and symptom burden, whereas the 5-point Likert scale for global satisfaction aligns with commonly validated summary outcome measures. Importantly, this checklist is a proposed framework rather than a validated instrument. It requires pilot testing, psychometric evaluation, and prospective validation across diverse immune-mediated disease populations before routine clinical implementation.

In rheumatology, integrating QoL measures complements and meaningfully extends the treat-to-target (T2T) strategy. While T2T focuses on achieving remission or low disease activity based on composite indices, QoL-oriented care incorporates patient-defined priorities such as pain control, fatigue reduction, functional independence, and maintenance of social roles (19). Improvements in body image, self-esteem, and social functioning following cosmetic interventions may be particularly relevant for patients with chronic inflammatory diseases, in whom visible manifestations contribute to stigma and diminished QoL. Emerging evidence indicates that routine integration of PROs into T2T frameworks enhances SDM, treatment adherence, and patient satisfaction, without compromising disease control.

## Clinical implications for aesthetic practice and shared decision-making

The incorporation of aesthetic medicine into the care of patients with IMDs carries important clinical implications. Large matched cohort studies and nationwide database analyses suggest that overall complication rates for many aesthetic and reconstructive procedures in patients with CTDs are comparable to those in the general population. Nevertheless, specific procedures, such as breast augmentation or abdominoplasty has been associated with increased risks of wound complications and venous thromboembolism in selected autoimmune subgroups (5, 18, 20).

These findings underscore the importance of structured pre-procedure assessment, including evaluation of disease stability, vascular status, wound-healing capacity, and prior complication history. Equally critical is the adoption of SDM frameworks to integrate PROs and safety perceptions into aesthetic care for individuals with IMDs. SDM facilitates bidirectional information exchange, whereby clinicians provide evidence-based risk assessments and procedural options, while patients express their values, expectations, and tolerance for uncertainty. Discussions among rheumatologists, dermatologists or plastic surgeons and patients support informed choices and individualized treatment planning.

From a rheumatology perspective, interdisciplinary collaboration and standardized patient education are essential to align aesthetic procedures with disease-specific considerations, strengthen patient trust, and reduce preventable adverse events. Grounding clinical decisions in both biomedical safety and patient-defined values, clinicians can provide aesthetic care that is technically sound, ethically responsible, and aligns with the principles of SDM.

## Future research agenda

Although evidence is increasing, long-term data on the safety and stability of aesthetic procedures in patients with IMDs remaines limited. Future research should prioritize longitudinal, prospective, multicenter studies incorporating standardized PROs measures and stratification by disease phenotype and immunomodulatory therapy. Given the heterogeneity of IMDs in immune pathways, organ involvement, and treatment regimens, safety perceptions and outcome priorities are likely to vary across subgroups. Comparative studies evaluating PROs among individuals receiving biologic versus targeted synthetic disease-modifying antirheumatic drugs may inform personalized risk counseling and procedural planning. Integration of real-world data sources and disease registries will further enhance external validity and clinical applicability.

Emerging trends underscore that immunosuppressive therapies may differentially modulate humoral and cellular immune responses, potentially shaping post-procedural risk profiles. Post-COVID immunomodulatory strategies may further affect safety perceptions, particularly in patients receiving biologics (21). Telemedicine potentially strengthens SDM in rheumatology; however, digital inequities may limit access for vulnerable groups, especially in rural areas (22). Transparent disclosure of any financial ties to the aesthetics industry remains essential.

Quality improvement studies assessing standardized pre-procedure assessment and education pathways may further reduce immune-related adverse events, including ASIA (10), and improve consistency of care. In summary, a research framework centered on PROs and safety perceptions represents a critical step toward responsible, patient-centered aesthetic care for individuals living with IMDs.

## Conclusion

Aesthetic and minimally invasive cosmetic procedures are increasingly pursued by patients with IMDs and can be performed safely in carefully selected individuals with stable disease. Existing evidence supports individualized risk assessment, immunologically informed procedural planning, and interdisciplinary collaboration. Awareness of procedure-specific risks, delayed immune-mediated reactions, and diagnostic uncertainties such as ASIA is essential. Clinicians should adopt structured, patient-centered decision-making to optimize safety, manage expectations, and minimize preventable complications in this unique population.
